# Persistent Cervicothoracic Muscle Mechanical Stiffness as a Potential Time-Dependent Mechanism Linking Postural Loading to Chronic Neck and Upper Thoracic Pain: A Narrative Review

**DOI:** 10.7759/cureus.112984

**Published:** 2026-07-19

**Authors:** Ammar Sawaf

**Affiliations:** 1 Acupuncture, University of Salford, Salford, GBR

**Keywords:** cervicothoracic muscle stiffness, chronic neck pain, forward-head posture, postural loading, shear-wave elastography, upper thoracic pain

## Abstract

Chronic neck and upper thoracic pain are common and frequently classified as non-specific when no single structural lesion explains the presentation. Although forward-head posture, prolonged device use, sedentary behaviour, and occupational loading are often considered contributors, the association between static posture and chronic neck pain is inconsistent. This narrative review examines persistent cervicothoracic muscle mechanical stiffness as a potential time-dependent mechanism linking cumulative postural loading to chronic symptoms. The central construct is persistent or repeatedly elevated mechanical stiffness, particularly passive stiffness assessed under standardised resting conditions; active stiffness related to ongoing activation or guarding is considered distinct but potentially relevant. PubMed, Scopus, and Web of Science were searched for English-language articles published from January 1990 to June 2026 addressing cervicothoracic posture, loading, fatigue, muscle mechanical properties, and chronic neck or upper thoracic pain. Findings were synthesised narratively without meta-analysis. Static posture alone does not consistently predict chronic neck pain. Sustained or repeated cervicothoracic loading may nevertheless increase muscular demand and contribute to fatigue, altered recruitment, impaired relaxation, reduced movement variability, and mechanical stiffness when exposure exceeds tissue tolerance and recovery capacity. Persistent mechanical stiffness has been proposed as a possible contributor to pain through mechanical sensitivity, altered perfusion relative to demand, metabolic stress, nociceptive sensitisation, and pain-related guarding. However, direct cervicothoracic evidence in patients with chronic neck pain remains limited; much of the biological rationale is extrapolated from studies of general muscle pain, myofascial pain, trapezius myalgia, and experimental models. In the absence of an identified neurological or vascular cause, alterations in neural-interface mechanics have been hypothesised to contribute to nerve-like symptoms; however, any relationship with surrounding muscle mechanical stiffness remains theoretical and has not been directly established. This review proposes a testable conceptual model in which cumulative postural loading, recovery capacity, neuromuscular adaptation, cervicothoracic muscle mechanical stiffness, and pain-related mechanisms interact over time. Objective assessment may help determine whether cervicothoracic muscle mechanical stiffness precedes pain, contributes to the proposed pathway, or identifies a clinically relevant subgroup. Direct longitudinal evidence validating this proposed pathway remains limited.

## Introduction and background

Chronic neck pain is a major global health problem and an important contributor to disability, reduced work productivity, healthcare utilisation, and impaired quality of life [1,2]. Neck pain frequently follows a recurrent or persistent course, and in many patients, symptoms cannot be fully explained by a single anatomical lesion or structural abnormality [3,4]. Such cases are commonly classified as non-specific neck pain, reflecting the multifactorial interaction of mechanical, neuromuscular, behavioural, occupational, and psychosocial factors [4,5].

Structural pathologies of the cervical spine, including disc herniation, foraminal stenosis, facet joint disease, fracture, inflammatory disorders, infection, malignancy, myelopathy, and clinically significant radiculopathy, remain important diagnostic considerations. However, degenerative cervical imaging findings are also common in asymptomatic individuals and do not consistently correlate with symptom severity [6,7]. Therefore, imaging findings should be interpreted in relation to clinical presentation, neurological examination, functional impairment, and potential non-structural contributors to pain.

Chronic neck pain may also involve altered nociceptive processing that cannot be explained solely by structural pathology or ongoing peripheral nociceptive input. Nociplastic pain describes pain arising from altered nociception when there is no clear evidence that actual or threatened tissue damage activating peripheral nociceptors, or a disease or lesion of the somatosensory system, fully explains the pain [8,9]. Central sensitisation refers to amplified neural signalling within the central nervous system that elicits pain hypersensitivity and may manifest clinically as hyperalgesia, allodynia, expanded pain distribution, or sensitivity beyond the primary symptomatic region [9]. Although related, nociplastic pain and central sensitisation are not synonymous, and neither should be assumed in every patient with chronic neck pain. Their relevance may vary among individuals and may interact with peripheral nociceptive input, sleep disturbance, stress, fear, pain-related beliefs, physical activity, and movement behaviour [8-10]. From a pain-neuroscience perspective, persistent symptoms are therefore best understood as emerging from interactions among peripheral tissue inputs, central pain processing, psychological and contextual factors, and behavioural responses rather than from a single structural, postural, or muscular abnormality [9].

Posture has long been considered a possible contributor to neck and upper thoracic pain. Forward-head posture, prolonged cervical flexion during mobile-device use, sustained sitting, occupational computer work, and thoracic flexion may increase mechanical demand on the cervical extensors, upper trapezius, levator scapulae, suboccipital muscles, thoracic extensors, and scapular stabilisers [11,12]. However, evidence linking static posture to neck pain and related symptoms is mixed. Some systematic evidence indicates that adults with neck pain may demonstrate greater forward-head posture and associations between selected postural measures, pain intensity, and disability [11]. In contrast, no significant association has been identified between forward-head posture and most neck-pain measures in adolescents [11]. Pooled findings also vary according to the postural measure examined: differences in craniovertebral angle have been reported between participants with and without neck pain, whereas pooled differences in forward-head posture and sagittal head angle were not statistically significant [10]. Occupational evidence similarly indicates that physical exposure or static posture alone does not consistently predict the development of non-specific neck pain among office workers [12].

Taken together, the available literature neither supports a uniformly consistent association between static posture and neck pain nor justifies dismissing posture as clinically irrelevant. Significant associations have been reported for selected postural measures and populations, particularly among adults, whereas other investigations have found no meaningful associations, especially in younger populations or when different postural variables and assessment methods are used [10,11]. These divergent findings may reflect differences in age, symptom characteristics, measurement methods, task conditions, and the postural construct being assessed. Moreover, a single observation of static posture represents only a momentary alignment and does not capture the duration and repetition of exposure, movement variability, occupational and task demands, muscular capacity and endurance, recovery opportunity, physical activity, sleep, stress, or other psychosocial and contextual factors. Consequently, two individuals with apparently similar forward-head posture may experience substantially different cumulative exposures and biological responses. Postural exposure should therefore be evaluated together with its duration and repetition, movement variability, occupational demands, muscular capacity, recovery, and psychosocial context. Accordingly, posture is considered in this review as a potential exposure characteristic whose clinical relevance depends on interactions among exposure dose, individual capacity, recovery, and broader biopsychosocial factors, rather than as either a universal cause of pain or a clinically irrelevant finding.

Within this broader pain-mechanism framework, persistent or repeatedly elevated cervicothoracic muscle mechanical stiffness may represent an under-investigated candidate mechanism linking cumulative postural loading to chronic pain in a subgroup of patients. In this review, muscle mechanical stiffness refers to resistance to deformation and is distinguished from perceived tightness and muscle spasm; passive and active stiffness are defined separately in the dedicated conceptual and operational definitions subsection. Sustained or repeated low-level muscular activity may promote fatigue, altered motor recruitment, impaired relaxation, reduced movement variability, and incomplete recovery. These responses may initially increase active stiffness through ongoing activation or co-contraction. With repeated exposure and inadequate recovery, alterations in passive mechanical stiffness may also develop in some individuals, although this temporal progression has not been established [13-16]. Persistent peripheral nociceptive input arising from mechanically sensitive muscle could plausibly interact with central pain amplification, while centrally mediated pain hypersensitivity, guarding, and altered motor behaviour could conversely influence perceived or objectively measured muscle stiffness [9]. These relationships may therefore be bidirectional and have not been established as a complete causal pathway in chronic neck pain. In selected patients, alterations in neural-interface mechanics have been hypothesised to contribute to nerve-like symptoms; however, any relationship with surrounding muscle mechanical stiffness remains theoretical and has not been directly established.

Despite growing literature concerning posture, occupational exposure, cervical muscle function, myofascial pain, and objective assessment of muscle mechanical properties, these domains have generally been investigated separately. Posture-focused research has predominantly examined static alignment or single-time-point associations with symptoms, whereas studies of muscle activation, fatigue, mechanical stiffness, microcirculation, and pain processing have employed heterogeneous constructs and measurement methods. Consequently, it remains unclear whether cumulative postural loading is associated with persistent alterations in cervicothoracic muscle mechanical behaviour, whether such alterations precede or follow pain, and whether they identify a clinically relevant subgroup. Distinctions among passive mechanical stiffness, active stiffness, muscle tone, perceived tightness, muscle spasm, and pain-related guarding have also been insufficiently articulated, limiting comparisons across studies and encouraging potentially reductionist interpretations. An integrative review is therefore needed to organise these adjacent but fragmented bodies of evidence, clarify the relevant constructs, identify the limitations of the proposed pathway, and establish testable priorities for prospective research.

**Figure 1:**
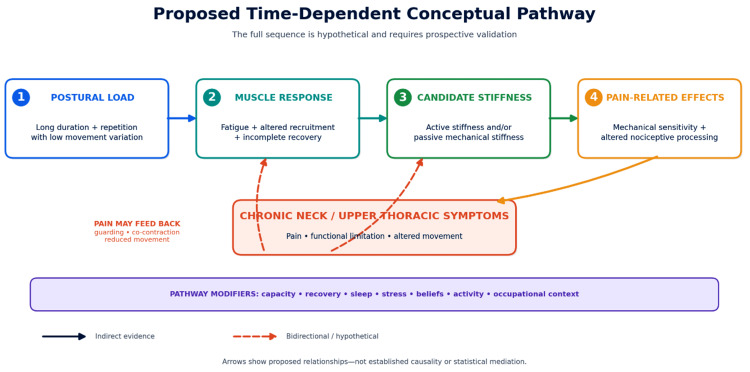
Proposed time-dependent conceptual pathway linking postural loading, muscle responses, cervicothoracic muscle mechanical stiffness, pain-related processes, and chronic symptoms. Solid arrows indicate indirectly supported forward relationships, whereas dashed arrows indicate hypothetical or bidirectional feedback from pain to muscle responses and stiffness. Capacity, recovery, sleep, stress, beliefs, activity, and occupational context may modify the proposed pathway. The figure is hypothesis-generating and does not establish causality or statistical mediation.

Accordingly, this narrative review re-examines posture-centric interpretations of chronic neck and upper thoracic pain by integrating evidence from posture research, occupational and cumulative loading, cervical biomechanics, neuromuscular control, muscle fatigue, myofascial pain mechanisms, objective assessment of muscle mechanical properties, central pain processing, nociplastic pain, and neural mechanosensitivity. It aims to: (1) clarify the conceptual and operational distinctions among passive mechanical stiffness, active stiffness, muscle tone, perceived tightness, guarding, and related constructs; (2) evaluate the biological plausibility and evidentiary limitations of persistent cervicothoracic muscle mechanical stiffness as a candidate link between cumulative postural loading and chronic symptoms; and (3) identify priorities for longitudinal and mechanistic research. The review proposes a testable conceptual model in which persistent or repeatedly elevated cervicothoracic muscle mechanical stiffness may represent a potential time-dependent mechanism linking cumulative postural exposure to chronic non-specific neck or upper thoracic pain (Figure 1). Central to this model is a load-capacity-recovery framework in which the potential significance of posture depends not on alignment alone but on whether cumulative exposure exceeds individual neuromuscular capacity and available recovery over time. This candidate mechanism is situated within a broader biopsychosocial and pain-neuroscience framework that recognises potential interactions among peripheral nociceptive input, central pain amplification, psychological and contextual factors, and pain-related behaviour [8,9]. The terms “potential mechanism” and “conceptual pathway” are used in a hypothesis-generating sense and do not imply that temporal precedence, causality, or a statistical mediation effect has been demonstrated.

## Review

Methodology

Given the conceptual and integrative objectives of this article, a narrative review design was adopted to synthesise heterogeneous evidence from posture research, occupational health, cervical biomechanics, neuromuscular physiology, myofascial pain, muscle-mechanical-property assessment, central pain processing, and clinical neck-pain literature. The review was designed to develop and critically examine a hypothesis-generating conceptual model rather than to provide an exhaustive systematic review, estimate pooled effect sizes, or determine quantitative intervention effects. Accordingly, no meta-analysis was planned or performed. The conduct and reporting of the review were informed by published methodological guidance for narrative literature reviews and by the quality domains described in the Scale for the Assessment of Narrative Review Articles (SANRA), including justification of the review’s importance, specification of its aims, description of the literature search, appropriate referencing, and balanced presentation of relevant evidence [17,18]. The SANRA was used as a quality-oriented framework rather than as a formal reporting guideline, numerical scoring instrument, or risk-of-bias tool.

Search Strategy

An electronic literature search was performed using PubMed, Scopus, and Web of Science databases to identify relevant English-language articles published between January 1990 and June 2026. The search strategy combined controlled vocabulary where available and free-text terms using Boolean operators. Search terms included combinations of the following: “chronic neck pain”, “non-specific neck pain”, “upper thoracic pain”, “forward head posture”, “cervical flexion”, “thoracic posture”, “sedentary behaviour”, “smartphone”, “computer work”, “cervical loading”, “upper trapezius”, “levator scapulae”, “suboccipital muscles”, “muscle stiffness”, “muscle tone”, “myotonometry”, “MyotonPRO”, “shear-wave elastography”, “electromyography”, “muscle fatigue”, “microcirculation”, “oxygenation”, “myofascial pain”, “trigger points”, “neural mechanosensitivity”, and “radicular symptoms”.

A representative PubMed search string was structured as follows: (“chronic neck pain” OR “non-specific neck pain” OR “upper thoracic pain”) AND (“forward head posture” OR “cervical flexion” OR “postural loading” OR “sedentary behaviour” OR “smartphone” OR “computer work”) AND (“muscle stiffness” OR “muscle tone” OR “muscle fatigue” OR “myotonometry” OR “shear-wave elastography” OR “electromyography”) AND (“microcirculation” OR “myofascial pain” OR “neural mechanosensitivity” OR “biomechanics”). Equivalent adaptations were applied for Scopus and Web of Science (See Appendix Table S1). A supplementary targeted search was also conducted to identify literature concerning central pain processing relevant to chronic neck and musculoskeletal pain. Search terms included combinations of “central sensitization”, “central sensitisation”, “nociplastic pain”, “pain hypersensitivity”, “hyperalgesia”, “allodynia”, “pain neuroscience”, and “pain neuroscience education”. Foundational consensus papers, narrative and systematic reviews, and clinically relevant studies were considered to contextualise the proposed peripheral muscle-mechanical pathway within a broader pain-neuroscience framework.

Eligibility Criteria

Studies were eligible for inclusion if they involved adult or adolescent populations with chronic, recurrent, or non-specific neck pain, upper thoracic pain, neck-shoulder pain, or asymptomatic comparison groups; examined posture, cervical or cervicothoracic loading, sedentary or device-related exposure, occupational factors, muscle activation, endurance, fatigue, stiffness, myofascial pain mechanisms, microcirculatory changes, or objective assessment of muscle mechanical properties; and reported findings with clinical, biomechanical, physiological, or methodological relevance to the proposed model. Eligible study designs included systematic reviews, meta-analyses, prospective cohort studies, cross-sectional studies, biomechanical studies, electrophysiological investigations, imaging studies, objective stiffness measurement studies, and mechanistic studies involving painful muscle. Studies were excluded if they focused exclusively on acute traumatic injury, fracture, infection, malignancy, inflammatory arthropathy, post-surgical conditions, paediatric neurological disorders, or cervical myelopathy without relevance to non-specific cervicothoracic pain or muscular mechanisms. Articles not available in English and publications without sufficient methodological or conceptual relevance were also excluded.

Study Selection Process

Titles and abstracts retrieved through the database searches were examined for relevance to the review objectives, and potentially relevant publications were assessed in full text. Priority was given to higher-level evidence where available, foundational mechanistic studies, methodologically relevant measurement studies, and literature representing both supportive and conflicting findings. Reference list screening and a supplementary targeted search were undertaken to identify literature concerning nociplastic pain, central sensitisation, pain neuroscience, and both significant and nonsignificant posture symptom associations. Study selection was undertaken by the sole author. Because the review was originally designed as a narrative rather than a systematic review, database-level screening counts and reasons for individual full-text exclusions were not prospectively recorded and could not be reconstructed reliably. Independent duplicate screening, protocol registration, inter-reviewer agreement assessment, and formal study-level risk-of-bias scoring were not performed. These limitations are acknowledged to avoid implying a level of systematic review methodology that was not undertaken.

Studies included in the illustrative studies table were selected from the literature considered in the narrative synthesis to illustrate the principal thematic domains of the review. Selection for the table was based on methodological relevance, directness to the specific thematic domain, inclusion of systematic or higher-level evidence where available, importance as a foundational mechanistic or measurement study, and capacity to represent supportive, mixed, or negative findings. Inclusion in the table did not indicate that a study directly validated the complete proposed pathway, and the table was not intended to provide an exhaustive inventory of all studies considered. Contradictory and null findings were considered during the narrative synthesis and were included in the table where they materially informed the interpretation of a thematic domain. Studies not displayed in the table continued to inform the text and were not excluded solely because their findings were inconsistent with the proposed model.

Data Extraction

Relevant information was extracted using a structured framework developed for this review. Extracted data included study design, population characteristics, exposure or assessment method, anatomical region examined, posture or loading variables, muscle activation or endurance findings, stiffness or tone measurement approach, microcirculatory or biochemical outcomes, pain and disability outcomes, and reported clinical or biomechanical interpretations. Particular attention was given to whether studies assessed static posture alone, cumulative exposure, muscular response, objective stiffness, or pain-related functional outcomes.

Narrative Synthesis

A thematic narrative synthesis was conducted. Evidence was organised into major domains concerning: (1) the inconsistent relationship between static posture and chronic neck pain; (2) cervicothoracic loading, muscular demand, and fatigue; (3) persistent muscle stiffness as a potential pain-generating or pain-maintaining state; (4) microcirculatory, metabolic, and nociceptive mechanisms; (5) objective assessment of muscle stiffness; and (6) possible biomechanical and neural-interface implications. Findings were interpreted according to consistency across studies, biological plausibility, methodological strength, and relevance to the proposed time-dependent model. No quantitative analysis or meta-analysis was performed because of heterogeneity in study populations, exposure definitions, measurement methods, and outcome variables.

Conceptual and Operational Definition of Muscle Mechanical Stiffness

The terminology used to describe muscle mechanical behaviour is heterogeneous, and terms such as stiffness, tone, tightness, guarding, and hardness should not be treated as interchangeable. In this review, mechanical stiffness is used as an overarching biomechanical construct referring to a muscle’s resistance to deformation in response to an applied force. This objective mechanical property is distinguished from subjective sensations of stiffness or tightness and from neural or behavioural responses to pain. Within this broader construct, an important distinction is made between passive and active stiffness. Passive stiffness refers to resistance to deformation when the muscle is relaxed and no voluntary contraction is intended. It reflects the combined contributions of muscle fibres, connective tissue, fascia, intramuscular pressure, and other non-contractile or viscoelastic components. Passive stiffness may be estimated using shear-wave elastography or mechanical myotonometry, provided that participant position, muscle length, contraction state, probe placement, and measurement procedures are standardised.

In contrast, active stiffness refers to resistance to deformation during voluntary or reflex muscle activation. It is influenced by contractile force, motor-unit recruitment, co-contraction, muscle length, and neural drive. Consequently, active stiffness may change with task demands, stabilisation requirements, fatigue, perceived threat, and pain and should not be considered equivalent to passive stiffness. Electromyography may provide complementary information regarding muscle activation, relaxation, co-contraction, and fatigue; however, it does not directly quantify either passive or active mechanical stiffness. Although related to these constructs, muscle tone has a distinct meaning. It refers broadly to the state of muscle tension or activation under resting conditions and may include neural, contractile, and intrinsic mechanical components, depending on the disciplinary and measurement context. Because tone is not synonymous with mechanical stiffness, the term is used in this review only when it reflects the terminology or outcome reported in the original study.

A further distinction is required between objectively measured mechanical properties and an individual’s subjective experience. Perceived stiffness or tightness refers to a self-reported sensation and does not necessarily correspond to objectively measured passive or active stiffness. An individual may report tightness without a demonstrable increase in mechanical stiffness, whereas objectively elevated stiffness may be present without a corresponding subjective sensation. Perceived stiffness is therefore considered a symptom-related construct rather than a direct measure of tissue mechanical behaviour. Similarly, guarding is understood as a protective neuromuscular response involving increased activation, co-contraction, movement restriction, or avoidance in response to actual or anticipated pain or threat. Guarding may increase active stiffness and may influence measurements obtained under nominally resting conditions, but it is not equivalent to passive mechanical stiffness. The term hardness is likewise treated cautiously because it is primarily descriptive or palpatory unless linked to a validated quantitative measurement; it is therefore not used as a substitute for mechanical stiffness. Against this conceptual background, the primary construct proposed for future empirical testing is persistent or repeatedly elevated cervicothoracic muscle mechanical stiffness, particularly passive stiffness assessed under standardised resting conditions. Active stiffness associated with persistent low-level activation, co-contraction, or guarding is considered a distinct but potentially interacting construct. The proposed model suggests that cumulative loading and inadequate recovery may be associated with sustained alterations in cervicothoracic muscle mechanical behaviour; however, the temporal progression from loading to active stiffness, passive stiffness, and pain has not been established. Accordingly, muscle mechanical stiffness is presented as a potential time-dependent mechanism requiring prospective investigation, rather than as a demonstrated causal mediator.

Results

The initial search identified 889 studies, and the final narrative synthesis incorporated 31 articles identified through database searching, reference list screening, and supplementary targeted searching. The evidence included systematic reviews and meta-analyses, observational studies, biomechanical and electromyographic investigations, painful muscle and myofascial studies, muscle-mechanical-property measurement studies, and conceptual or clinical reviews. Table 1 presents illustrative studies selected to represent the principal thematic domains identified in the narrative synthesis, including supportive, mixed, null, indirect, and hypothesis-generating evidence. The studies were selected for their methodological relevance, contribution to a specific component of the proposed model, or importance as foundational or measurement research. The table is not an exhaustive list of all literature considered, and inclusion does not indicate that a study directly validates the complete proposed pathway. Contradictory and negative findings were incorporated where they materially informed interpretation, particularly in relation to posture-pain associations. Because the available studies generally examine individual components of the proposed pathway rather than the complete temporal sequence from cumulative loading to mechanical stiffness and subsequent pain, the directness of each evidence domain is classified explicitly in the table. Where quantitative or mechanistic findings are discussed, they are interpreted descriptively and were not pooled because of heterogeneity in study design, populations, exposure definitions, measurement methods, and outcome variables.

**Table 1:** Illustrative Studies Representing Supportive, Mixed, Indirect, and Hypothesis-Generating Evidence across the Major Themes of the Narrative Synthesis EC = evidence classification; DE = direct evidence; IE = indirect evidence; HGE = hypothesis-generating evidence; ME = mixed evidence; EMG = electromyographic. Note: Studies were selected to illustrate the principal thematic domains of the narrative synthesis and were not intended to constitute an exhaustive list of all literature considered. Selection reflected methodological relevance, contribution to a specific component of the conceptual model, inclusion of higher-level or foundational evidence where available, and representation of supportive, mixed, or null findings. “Direct evidence” refers to longitudinal research directly testing the complete pathway from cumulative postural loading through cervicothoracic muscle mechanical stiffness to chronic pain. “Indirect evidence” refers to studies examining one or more components without testing the complete temporal sequence. “Hypothesis-generating evidence” refers to conceptual, mechanistic, measurement, or adjacent-domain research that supports plausibility or testability. No included study directly tested the complete proposed pathway using a longitudinal design and formal mediation analysis.

Core thematic domain	Illustrative studies	EC	Study design	Population/setting	Key findings relevant to chronic neck and upper thoracic pain
Chronic neck pain as a multifactorial condition	Guzman et al. [4]; Blanpied et al. [5]	IE	Conceptual model; clinical practice guideline	Patients with neck pain	Neck pain is best understood through interaction among structural, functional, psychological, occupational, and social factors rather than a single anatomical lesion.
Cervical imaging findings and symptom discordance	Matsumoto et al. [6]; Nakashima et al. [7]	IE	MRI observational studies	Asymptomatic individuals	Degenerative cervical findings are common in people without symptoms, supporting cautious interpretation of imaging findings.
Nociplastic pain and central sensitisation	Kosek et al. [8]; Nijs et al. [9]	IE	Clinical criteria and grading framework; narrative or state-of-the-art review	Chronic musculoskeletal and spinal pain contexts.	Nociplastic pain and central sensitisation may be relevant in selected patients with chronic musculoskeletal pain and may interact with peripheral nociceptive, psychological, contextual, and behavioural factors.
Forward-head posture and neck pain	Rani et al. [10]; Mahmoud et al. [11]	IE/ME	Systematic review and meta-analysis	Adults and adolescents	Selected postural measures may be associated with symptoms in some populations, but null findings are common; results depend on measurement and population characteristics, and static posture alone is insufficient to explain pain.
Occupational and postural exposure	Jun et al. [12]; Hansraj [19]; Huysmans et al. [20]	IE	Systematic review/meta-analysis; prospective occupational study	Office workers and working populations	Physical exposure, perceived muscular tension, and work-related factors may contribute to neck symptoms, but posture alone does not fully explain pain development.
Sustained low-level loading and occupational myalgia	Hägg [14]; Jensen et al. [21]	IE	Mechanistic model; occupational EMG study	Occupational shoulder-neck complaints	Prolonged low-level muscle activity may contribute to fatigue, local overload, and shoulder-neck symptoms.
Muscle activation and endurance alterations	Falla et al. [13]; O’Leary et al. [22]; Edmondston et al. [23]	HGE	Electromyographic and clinical studies	Patients with chronic or postural neck pain	Chronic neck pain is associated with altered cervical muscle activation, reduced deep cervical flexor performance, and impaired endurance.
Myofascial biochemical and nociceptive mechanisms	Shah et al. [15,16]; Larsson et al. [24]; Rosendal et al. [25]	IE	Microdialysis and physiological studies	Painful muscle/myalgia populations	Painful muscles may demonstrate altered biochemical, metabolic, blood-flow, or nociceptive mediator profiles.
Objective assessment of muscle stiffness	Agyapong-Badu et al. [26]; Kelly et al. [27]	HGE	Reliability and measurement studies	Healthy or musculoskeletal populations	Myotonometry and shear-wave elastography can quantify mechanical properties of muscle, although neck-specific thresholds remain insufficiently established.
Neural-interface and nerve-like symptoms	Carette and Fehlings [28]; Coppieters and Butler [29]	HGE	Clinical review; neurodynamic analysis	Cervical radiculopathy and neural mechanosensitivity contexts	Nerve-like symptoms require careful neurological assessment; non-compressive neural mechanosensitivity may contribute in selected cases.

The Inconsistent Relationship Between Static Posture and Chronic Neck Pain

The literature demonstrates that the relationship between static posture and chronic neck pain is not straightforward. Forward-head posture and sustained cervical flexion are biomechanically plausible contributors to increased muscular demand, particularly in the posterior cervical and upper thoracic regions. However, systematic evidence indicates that posture alone does not consistently predict pain across populations [11,12]. Adults with neck pain may demonstrate greater forward-head posture than asymptomatic adults, but this association is less consistent in adolescents and younger populations [11]. Moreover, null findings have been reported for several posture-symptom comparisons, and pooled conclusions differ according to the postural variable examined; therefore, evidence concerning one measure, such as craniovertebral angle, should not be generalised to all forms of head, cervical, or thoracic posture [10]. These findings suggest that a single postural measure may be insufficient to explain chronic pain development. A key limitation of posture-based research is that most studies assess visible alignment at one point in time rather than cumulative exposure. Static posture does not capture duration of loading, repetition, recovery opportunity, physical activity history, muscular endurance, sleep, stress, occupational demand, or previous symptoms. Therefore, the current posture may poorly represent the biological loading history of cervicothoracic tissues. This may explain why some individuals with forward-head posture remain asymptomatic, whereas others develop persistent symptoms.

Cervicothoracic Loading, Muscle Demand, and Fatigue

Biomechanical and occupational evidence suggests that sustained cervical flexion, prolonged sitting, mobile device use, computer work, and low task variation may increase demand on cervicothoracic muscles [12,19-21]. The cervical extensors, upper trapezius, levator scapulae, suboccipital muscles, thoracic extensors, and scapular stabilisers may be required to maintain head, neck, thoracic, and shoulder-girdle positions for prolonged periods. Although these tasks generally involve low levels of force, sustained low-level muscle activity may contribute to fatigue, altered recruitment, and reduced relaxation in susceptible individuals [14,21].

Studies involving patients with chronic neck pain have reported altered neuromuscular behaviour, including reduced deep cervical flexor performance, altered superficial muscle activation, impaired endurance, and changes in motor-control strategies [13,22,23]. However, most of this evidence is cross-sectional or mechanistic and does not establish temporal ordering. In particular, direct prospective evidence demonstrating that cumulative cervicothoracic loading produces persistent alterations in muscle mechanical stiffness that subsequently lead to chronic pain remains limited. The available findings therefore support biological plausibility and the formulation of a testable pathway but do not demonstrate that loading-related muscle dysfunction precedes pain or acts as a causal intermediate mechanism.

These muscle-function findings should also be interpreted within a broader biopsychosocial and pain-neuroscience framework. Occupational demands, task duration, movement variability, muscular capacity, physical activity, sleep, stress, pain-related beliefs, fear, and recovery opportunities may influence both muscular responses to loading and the experience of pain. Similarly, peripheral nociceptive input may interact with central pain amplification, while pain-related threat, guarding, and altered movement behaviour may contribute to co-contraction, reduced movement variability, fatigue, and perceived or objectively measured stiffness [9]. These relationships are likely to be reciprocal rather than unidirectional.

Accordingly, the observed alterations in muscle activation, endurance, and motor control should not be interpreted as evidence that muscle dysfunction is invariably the primary cause of chronic neck pain. They indicate that muscle function may represent one potentially relevant component of a heterogeneous clinical presentation. Muscular mechanisms should therefore be evaluated alongside cumulative exposure, structural and neurological findings, pain processing characteristics, psychological and contextual factors, occupational demands, and recovery capacity. Longitudinal studies combining objective measures of exposure, muscle activation, passive and active mechanical stiffness, pain sensitivity, and psychosocial variables are required to determine the temporal and potentially bidirectional relationships among loading, muscle dysfunction, and chronic pain.

Persistent Alterations in Muscle Mechanical Stiffness as a Potential Pain-Related State

The literature considered in this review provides predominantly indirect and hypothesis-generating evidence that altered muscle mechanical behaviour may represent a plausible but insufficiently tested candidate mechanism linking cumulative postural exposure with chronic pain. Temporary increases in active stiffness may occur during stabilisation, task performance, protective co-contraction, or pain-related guarding. The clinically relevant hypothesis considered in this review is that repeated loading with incomplete recovery may be associated with persistent or repeatedly elevated mechanical stiffness, including passive stiffness measured under standardised resting conditions. However, the available evidence does not establish that active stiffness necessarily progresses to altered passive stiffness, that either construct precedes chronic pain, or that mechanical stiffness mediates the association between cumulative postural loading and subsequent symptoms.

Persistent or repeatedly elevated mechanical stiffness has been proposed as a possible contributor to symptoms through several interacting mechanisms. Increased mechanical stiffness may be associated with local mechanical sensitivity, reduced movement variability, altered proprioceptive input, and the concentration of loading through restricted movement patterns. Painful muscles may also demonstrate altered local biochemical and physiological characteristics, including changes in blood flow, oxygenation, metabolic activity, inflammatory mediators, and nociceptive substances [15,16,24,25]. These findings provide indirect biological plausibility for a possible muscle-related contribution to pain but do not establish that postural loading causes these changes, that mechanical stiffness precedes them, or that they have been demonstrated specifically as a complete pathway in cervicothoracic muscles of patients with chronic neck pain.

Importantly, altered mechanical stiffness may be both a contributor to and a consequence of pain. Pain can induce guarding, co-contraction, sympathetic arousal, and reduced movement, which may increase active stiffness and influence measurements obtained under nominal resting conditions. Perceived tightness may also occur without objectively elevated mechanical stiffness. Longitudinal studies are therefore required to determine whether objectively measured passive or active stiffness precedes pain, follows pain, or identifies a particular clinical subgroup.

Measurement of Cervicothoracic Muscle Stiffness

Objective measurement methods are increasingly relevant for testing the proposed model, although the available tools assess different dimensions of muscle behaviour and should not be treated as interchangeable. Myotonometry characterises the mechanical response of superficial tissue to a brief external impulse and may provide device-specific estimates of dynamic stiffness, oscillation frequency or resting tone, elasticity, relaxation, and creep. Shear-wave elastography estimates region-specific tissue mechanical behaviour through shear-wave propagation, commonly reported as shear-wave speed or an estimated elastic modulus. Both methods are influenced by participant position, muscle length and activation, probe or transducer placement, tissue depth, fibre orientation, device settings, and measurement protocol [26,27].

Electromyography provides complementary information regarding electrical muscle activation, co-contraction, relaxation, and fatigue, but it does not directly measure passive or active mechanical stiffness. Likewise, clinical palpation provides an examiner-dependent impression of tissue resistance, tension, tenderness, or hardness rather than an objective measure of mechanical stiffness. Perceived stiffness or tightness is a patient-reported sensation that may reflect pain, fatigue, guarding, threat, or altered sensory processing and may not correspond to instrumentally measured stiffness. Myotonometric outcomes, elastographic outcomes, electromyographic activity, palpated resistance, and perceived stiffness should therefore be interpreted as related but distinct constructs. Preliminary reference values have been reported for selected neck and shoulder muscles and specific shear-wave elastography protocols in healthy participants. However, these findings are based on limited samples and are affected by factors such as the muscle examined, sex, participant characteristics, contraction state, measurement orientation, device, and acquisition protocol. Consequently, sufficiently validated and generalizable neck-specific normative ranges, diagnostic cut-offs, and clinically meaningful change thresholds remain insufficiently established.

Ultrasound and MRI may provide complementary structural information, including muscle morphology, cross-sectional area, thickness, and fatty infiltration, but these findings do not directly quantify muscle mechanical stiffness. Therefore, objective assessment of cervicothoracic muscle mechanical properties should currently be considered primarily a research, longitudinal monitoring, and phenotyping approach rather than a standalone diagnostic test. An isolated stiffness value should not be used to determine the cause of an individual patient’s neck pain.

Biomechanical and Neural-Interface Implications

Muscles not only move the cervical and thoracic spine; they also contribute to stabilisation, compression, and segmental load distribution. Persistent muscle activity, co-contraction, or stiffness may therefore modify cervicothoracic biomechanics by increasing compressive demand, reducing movement variability, and altering load sharing between active and passive structures. However, current evidence does not justify the conclusion that muscle stiffness directly causes cervical disc degeneration, loss of disc height, or structural narrowing. These possibilities should remain hypotheses requiring longitudinal imaging and biomechanical validation.

Some patients with chronic neck or upper thoracic pain report nerve-like symptoms, including radiating pain, paresthesias, tingling, burning pain, heaviness, weakness, or altered sensation. These symptoms should not be attributed to muscle mechanical stiffness without appropriate clinical assessment and should prompt evaluation to exclude cervical radiculopathy, myelopathy, peripheral neuropathy, vascular pathology, and other neurological or serious causes [28]. In selected patients without evidence of nerve-root compression or another neurological or vascular disorder, altered neural mechanosensitivity or changes in the mechanical interface surrounding peripheral nerves may contribute to symptoms [29]. Persistent or repeatedly elevated stiffness in cervicothoracic or shoulder-girdle muscles has been hypothesised to influence neural-interface mechanics; however, this relationship remains theoretical and has not been shown to produce nerve compression or neural symptoms.

Discussion

This narrative review re-examined the relationship between cervicothoracic posture, cumulative postural loading, persistent muscle stiffness, and chronic neck or upper thoracic pain. The evidence synthesised in this review suggests that static posture alone is unlikely to provide a sufficient explanation for chronic pain development. However, this does not necessarily mean that posture is clinically irrelevant. A more plausible interpretation is that posture may act as an exposure state whose biological significance depends on duration, repetition, muscular demand, movement variability, tissue capacity, and recovery. Within this framework, persistent or repeatedly elevated cervicothoracic muscle mechanical stiffness may represent a potential time-dependent mechanism linking cumulative loading to chronic symptoms.

The inconsistent association between forward-head posture and neck pain is one of the central findings of the literature. Although adults with neck pain may show greater forward-head posture in some pooled analyses, this relationship is not consistently observed across all populations, particularly in adolescents [11]. Similarly, occupational studies suggest that physical exposure contributes to neck pain risk but does not fully explain symptom development when considered in isolation [12]. These findings challenge a simplistic posture-centric model in which a visible alignment abnormality is assumed to directly cause pain. Instead, they support a broader interpretation in which posture becomes clinically meaningful only when considered together with exposure dose, task duration, muscular endurance, recovery opportunity, and individual susceptibility.

A major limitation of static posture research is that it often captures only a brief moment of alignment. Such an assessment cannot determine how long a posture has been sustained, how frequently it recurs, whether the individual varies position, or whether the involved tissues have developed fatigue or stiffness over time. This may explain why some individuals with apparently similar forward-head posture remain asymptomatic, whereas others develop persistent neck or upper thoracic symptoms. Accordingly, the clinically relevant question may not be whether a posture is inherently “good” or “bad”, but whether the duration and repetition of postural exposure, considered together with movement variability, individual capacity, recovery, and psychosocial context, exceed the adaptive capacity of the cervicothoracic neuromuscular system.

The proposed stiffness-related conceptual pathway is biologically plausible and indirectly supported by several converging lines of evidence, although these studies do not demonstrate the complete causal sequence from postural exposure to mechanical stiffness and chronic pain. Biomechanically, forward displacement of the head and sustained cervical flexion can increase the flexion moment acting on the cervical spine and may increase demand on posterior cervical and upper thoracic muscles [19]. Occupational and electromyographic studies indicate that prolonged low-level muscle activity may contribute to fatigue, altered recruitment, and shoulder-neck complaints [14,20,21]. Clinical studies also demonstrate altered cervical muscle activation, reduced deep cervical flexor performance, impaired endurance, and altered motor-control strategies in patients with chronic neck pain [13,25,26]. Collectively, these findings indicate that functional neuromuscular alterations are associated with chronic neck pain and may be clinically relevant, but they do not establish that muscle dysfunction precedes pain or represents the primary cause in individual patients.

Persistent or repeatedly elevated muscle mechanical stiffness has been proposed as a possible contributor to pain through interacting mechanical and physiological processes. Mechanically, altered stiffness may be associated with reduced movement variability, sensitivity to stretch or compression, altered proprioceptive input, and concentration of physical loading through restricted movement patterns. Studies of painful muscle, myofascial pain, and trapezius myalgia have reported alterations in local blood flow, oxygenation, metabolic activity, and concentrations of nociceptive or inflammatory substances [15,16,24,25]. However, these findings provide indirect biological plausibility rather than direct evidence that cervicothoracic muscle mechanical stiffness causes reduced perfusion or metabolic changes in patients with chronic neck pain. It remains unclear whether altered perfusion and metabolic findings precede mechanical stiffness, arise from sustained muscle activation, follow pain-related guarding, or reflect interacting processes. The available evidence, therefore, does not establish a complete causal sequence from postural loading to mechanical stiffness, altered perfusion or metabolism, and chronic pain. These relationships may be bidirectional because pain can contribute to guarding, co-contraction, reduced movement, and changes in perceived or objectively measured stiffness.

The evidence underlying the proposed model can therefore be considered at three levels. Demonstrated findings include associations of chronic neck pain with altered muscle activation, impaired endurance, motor-control changes, and selected painful muscle physiological characteristics. Inferred mechanisms include the possibility that cumulative loading and incomplete recovery contribute to persistent changes in active or passive mechanical stiffness and that these changes interact with pain-related processes. Hypothetical mechanisms include the complete temporal sequence from postural exposure to persistent mechanical stiffness and subsequent chronic pain, as well as stiffness-related effects on perfusion, structural degeneration, or neural-interface mechanics. The inferred and hypothetical components remain to be tested prospectively and should not be interpreted as established mechanisms in patients with chronic cervicothoracic pain. These constructs should nevertheless remain conceptually distinct. Passive mechanical stiffness refers to resistance to deformation under standardised resting conditions, whereas active stiffness reflects the contribution of voluntary or involuntary muscle activation. Guarding is a protective motor behaviour that may increase active stiffness, while perceived stiffness or tightness is a subjective experience that may not correspond to either construct. Consequently, evidence concerning tone, activation, guarding, perceived tightness, or palpated hardness cannot be treated as direct evidence of altered passive mechanical stiffness. Peripheral muscle-related mechanisms should also be interpreted in relation to central pain processing. In some individuals with chronic neck pain, peripheral nociceptive input, altered pain modulation, and central sensitisation may interact with sleep disturbance, stress, fear, hypervigilance, and pain-related movement behaviour [9]. Conversely, centrally amplified pain and perceived threat may contribute to guarding, co-contraction, reduced movement variability, and perceived stiffness. Accordingly, objectively measured muscle mechanical stiffness, perceived tightness, and pain hypersensitivity should be evaluated as related but distinct constructs. The proposed model does not assume that chronic neck pain is universally nociplastic or centrally mediated; rather, nociplastic features and central sensitisation may characterise a subgroup and may coexist with peripheral nociceptive and mechanical contributors [8,9].

The proposed model also helps explain why posture correction alone may be insufficient for many patients. If persistent stiffness, fatigue, impaired relaxation, and reduced load tolerance have already developed, simply instructing a patient to sit upright may not resolve the underlying neuromuscular state. In some cases, rigid postural correction may even increase muscular effort or pain-related vigilance. A more clinically appropriate approach may be to emphasise movement variability, graded exposure, muscular endurance, relaxation capacity, ergonomic load reduction, and recovery. This interpretation is consistent with contemporary guideline-based approaches that discourage overly structural explanations and favour active, functional, and biopsychosocial management of chronic neck pain [5].

The biomechanical implications of persistent stiffness require cautious interpretation. Cervicothoracic muscles contribute not only to movement but also to stabilisation and spinal loading. Increased muscle activity or co-contraction may increase compressive forces and alter load distribution across cervical and upper thoracic segments. Therefore, it is mechanically plausible that persistent stiffness may modify cervicothoracic loading. However, current evidence does not justify stating that muscle stiffness directly causes cervical disc degeneration, loss of disc height, foraminal narrowing, or structural compression. Degenerative cervical findings are common in asymptomatic individuals and must be interpreted within the broader clinical context [6,7]. Thus, stiffness should be viewed as a potential functional and biomechanical contributor rather than as a proven structural cause.

The same cautious reasoning applies to nerve-like symptoms. Patients with chronic neck or upper thoracic pain may report radiating pain, tingling, burning, heaviness, or altered sensation. Such symptoms require careful neurological assessment to exclude cervical radiculopathy, myelopathy, peripheral neuropathy, vascular pathology, or other serious causes [28]. In some patients without clear nerve-root compression, altered neural mechanosensitivity or impaired neural-interface mechanics may contribute to symptoms [29]. Persistent stiffness in the scalene, pectoral, upper trapezius, levator scapulae, or cervicothoracic paraspinal regions may theoretically affect the mechanical environment around neural structures. Nevertheless, it would be premature to claim that stiff muscles definitively “trap” nerves. The more defensible position is that stiffness may contribute to neural-interface sensitivity in selected patients, and this hypothesis requires direct testing.

The proposed stiffness-related model should be interpreted alongside, rather than in competition with, other models and frameworks relevant to chronic pain. A posture-centric model emphasises visible alignment and mechanical strain, whereas the present model emphasises possible tissue and neuromuscular responses to cumulative exposure in relation to individual capacity and recovery. Central sensitisation and nociplastic pain perspectives emphasise altered nociceptive processing and pain hypersensitivity, although these related concepts are not synonymous and should not be assumed in every patient. Fear-avoidance models emphasise threat appraisal, pain-related fear, vigilance, avoidance, and subsequent disability. Motor-adaptation models propose that pain may alter muscle recruitment and movement strategies, potentially contributing to guarding, co-contraction, reduced movement variability, and changes in active stiffness. The biopsychosocial framework provides the overarching context within which these peripheral, central, psychological, behavioural, occupational, and social influences may interact. Accordingly, the proposed mechanical stiffness pathway is best viewed as one potentially relevant component within a broader biopsychosocial formulation rather than as a competing or comprehensive explanation of chronic neck pain. Table 2 retains the original comparison between posture-centric and stiffness-related interpretations while extending it to these complementary perspectives, comparing their primary emphasis, potential explanatory contribution, principal limitations, and relationship to the proposed stiffness-related pathway.

**Table 2:** Comparison of complementary conceptual models and frameworks relevant to chronic neck and upper thoracic pain. Note: The models and frameworks are presented as complementary rather than mutually exclusive. The first two rows retain the original comparison between posture-centric and stiffness-related load-capacity-recovery interpretations. The additional rows situate the proposed pathway within central pain-processing, fear-avoidance, motor-adaptation, and biopsychosocial perspectives. The table does not imply that any single model provides a complete explanation of chronic neck or upper thoracic pain or that the proposed stiffness-related pathway has been causally established.

Model or framework	Primary emphasis	Potential explanatory contribution	Principal limitation	Relationship to the proposed stiffness-related pathway
Posture-centric model	Visible alignment or static posture	Identifies posture as a possible mechanical exposure characteristic	Static posture alone does not capture exposure duration, repetition, movement variability, individual capacity, recovery, or pain processing	Provides a potential exposure component, but does not explain the complete proposed pathway
Stiffness-related load-capacity-recovery model	Possible tissue and neuromuscular responses to cumulative loading and incomplete recovery	Proposes potentially measurable changes in active and passive muscle mechanical stiffness	The proposed temporal and causal pathway remains unestablished	Represents the central hypothesis evaluated in this review
Central-sensitisation/nociplastic-pain model	Altered nociceptive processing and pain hypersensitivity	May help explain disproportionate, widespread, or persistent symptoms in selected patients	Central sensitisation or nociplastic pain should not be assumed in every chronic neck-pain presentation	May interact bidirectionally with peripheral nociceptive input, guarding, motor behaviour, and perceived stiffness
Fear-avoidance model	Threat appraisal, pain-related fear, vigilance, avoidance, and disability	Explains how beliefs and behaviour may contribute to symptom persistence, activity restriction, and disability	Does not independently establish alterations in tissue mechanical properties	Fear and vigilance may contribute to guarding, co-contraction, reduced movement, and perceived stiffness
Motor-adaptation model	Pain-related redistribution of muscle activity and movement strategies	Helps explain altered recruitment, co-contraction, impaired relaxation, and reduced movement variability	Adaptations vary among individuals and may be protective, neutral, or maladaptive	May influence active stiffness, fatigue, movement variability, and loading distribution
Biopsychosocial framework	Interactions among biological, psychological, occupational, behavioural, and social factors	Provides an overarching framework for understanding heterogeneous chronic pain presentations	It is a broad integrative framework rather than a specific tissue-level mechanism	Encompasses the proposed stiffness-related pathway and the other complementary models

The clinical implications of this model are important. First, clinicians should avoid deterministic statements suggesting that forward-head posture inevitably causes chronic neck pain. Such statements may increase fear, vigilance, and unnecessary concern. Second, clinicians should also avoid dismissing posture entirely, because sustained or repeated loading may still be relevant when considered as part of cumulative exposure. Third, assessment should include not only structural findings and visible posture but also muscle stiffness, endurance, motor control, task exposure, sleep, stress, movement habits, physical activity, and recovery opportunity. Fourth, treatment should ideally target modifiable mechanisms, including excessive sustained loading, reduced movement variability, poor endurance, persistent guarding, and inadequate recovery. For patients demonstrating pain hypersensitivity, fear, or unhelpful structural beliefs, individualised pain neuroscience education may complement active rehabilitation, although it should not replace appropriate physical, neurological, and psychosocial assessment [30].

Objective measurement is central to advancing this hypothesis. Myotonometry and shear-wave elastography may help quantify muscle mechanical properties, while electromyography can evaluate activation, relaxation, co-contraction, and fatigue behaviour [26,27]. Ultrasound, MRI, endurance testing, pressure-pain thresholds, wearable posture monitoring, and functional movement analysis may provide complementary information [31,32]. Future studies should avoid relying solely on static posture photographs or single-time-point alignment measures. Instead, longitudinal designs should assess whether cumulative exposure predicts later stiffness, whether stiffness predicts pain onset or persistence, and whether reducing stiffness improves symptoms independently of changes in visible posture. The proposed time-dependent model also raises questions relevant to prevention, but its implications for children and adolescents remain uncertain. Existing evidence concerning posture, device use, sedentary behaviour, movement variability, cervicothoracic mechanical stiffness, and subsequent pain in younger populations is predominantly cross-sectional or indirect, and posture-pain associations are less consistent in adolescents than in adults [11,33-35]. It therefore cannot currently be concluded that forward-head or flexed postures, device use, or low movement variability produce progressive cervicothoracic muscle mechanical stiffness or predict later chronic pain in children and adolescents. Preventive recommendations should consequently avoid fear-based messages, rigid posture correction, or implying that a particular posture is inherently harmful. General encouragement of regular movement, task variation, physical activity, adequate recovery, and symptom-responsive load management may be reasonable for overall musculoskeletal health, but these recommendations should not be presented as proven methods for preventing stiffness-mediated neck pain. Prospective studies are required to determine whether cumulative exposure, movement variability, recovery patterns, and objectively measured mechanical stiffness are associated with subsequent neck or upper thoracic pain in younger populations. Furthermore, future investigations should prespecify whether passive stiffness, active stiffness, muscle activation, muscle tone, or perceived stiffness is being evaluated and should avoid treating these outcomes as interchangeable. Prospective studies should standardise participant position, muscle length, contraction state, measurement site, device settings, time of assessment, recent activity, and symptom status. Adequately powered longitudinal designs with formal mediation analyses would be required before cervicothoracic muscle mechanical stiffness could be described as a mediator of the relationship between cumulative postural loading and subsequent pain.

This review has some limitations. As a narrative review, literature selection and interpretation may have been influenced by author judgement, and no formal risk-of-bias assessment or meta-analysis was performed. The terminology surrounding stiffness, tone, tightness, hardness, guarding, and myofascial pain is inconsistent, and different studies may assess different tissue, neural, perceptual, or behavioural properties. Direct evidence linking cumulative postural exposure to objectively measured cervicothoracic muscle mechanical stiffness and subsequent chronic pain remains limited. Evidence concerning altered perfusion, oxygenation, and metabolic processes is derived largely from adjacent research involving painful muscle, myofascial pain, trapezius myalgia, and mechanistic models rather than direct evaluation of the complete proposed pathway in chronic cervicothoracic pain. These studies support biological plausibility but do not demonstrate that muscle mechanical stiffness produces perfusion or metabolic abnormalities. Although the proposed pathway is conceptually plausible and empirically testable, its temporal sequence, causal relationships, and mediation effect have not been established. In addition, mechanical stiffness may be a contributor to or consequence of pain, and bidirectional relationships are likely. Despite these limitations, the proposed model offers a clinically and scientifically useful framework. It helps reconcile conflicting posture-pain findings by suggesting that posture alone may be an insufficient predictor unless the intervening biological response is measured. Persistent or repeatedly elevated cervicothoracic muscle mechanical stiffness may represent one candidate response. If future longitudinal and mechanistic studies confirm this pathway, the management of chronic neck and upper thoracic pain may shift further away from simple posture correction and towards preserving movement variability, improving muscular endurance, enhancing recovery, reducing unnecessary guarding, and identifying objectively measurable stiffness-related subgroups.

## Conclusions

Chronic neck and upper thoracic pain should not be explained solely by visible posture or isolated structural findings. Although forward-head posture, prolonged cervical flexion, sedentary behaviour, and occupational loading may increase cervicothoracic muscular demand, current evidence does not support posture as an inevitable or standalone cause of chronic pain. This narrative review proposes a conceptual model in which persistent or repeatedly elevated cervicothoracic muscle mechanical stiffness may represent a potentially measurable time-dependent mechanism linking cumulative postural loading to chronic non-specific neck or upper thoracic pain. This hypothesised pathway has not been causally demonstrated. The central construct proposed for empirical testing is objectively measured mechanical stiffness, particularly passive stiffness assessed under standardised resting conditions. Active stiffness associated with continued activation, co-contraction, or guarding is considered a related but distinct construct, while perceived tightness should be treated as a subjective symptom rather than an equivalent biomechanical measure. The proposed pathway may involve interactions among sustained low-level loading, fatigue, altered motor recruitment, impaired relaxation, inadequate recovery, active and passive mechanical stiffness, mechanical sensitivity, altered perfusion relative to demand, metabolic changes, nociceptive sensitisation, and pain-related guarding. However, the temporal ordering and causal relationships among these factors remain uncertain. Direct evidence concerning perfusion and metabolic mechanisms in cervicothoracic pain populations is limited, and mechanical stiffness may be a contributor to, consequence of, or correlate of pain. This model does not suggest that muscle mechanical stiffness explains all chronic neck pain or that it has been demonstrated to cause disc degeneration, structural narrowing, nerve compression, nerve entrapment, or neurological symptoms. Rather, persistent or repeatedly elevated cervicothoracic muscle mechanical stiffness should be investigated as a potentially measurable and modifiable candidate mechanism within a broader biopsychosocial and pain-neuroscience framework. Prospective longitudinal studies incorporating objective measurements of cumulative postural exposure, active and passive mechanical stiffness, pain and functional outcomes, pain sensitivity, and relevant psychosocial factors are required to validate the proposed pathway and establish temporal ordering. Formal mediation analyses would be required before cervicothoracic muscle mechanical stiffness could be considered a demonstrated mediator between cumulative postural loading and chronic pain.

## Appendices

Table S1 

**Table 3:** Search strategies and search term. Note: TITLE-ABS-KEY: title, abstract, and keyword search; TS: topic search.

Database	Search period	Search terms
PubMed	January 1990 to June 2026	(“chronic neck pain” OR “non-specific neck pain” OR “upper thoracic pain”) AND (“forward head posture” OR “cervical flexion” OR “thoracic posture” OR “postural loading” OR “sedentary behaviour” OR “smartphone” OR “computer work”) AND (“muscle stiffness” OR “muscle tone” OR “muscle fatigue” OR “myotonometry” OR “MyotonPRO” OR “shear-wave elastography” OR “electromyography”) AND (“microcirculation” OR “oxygenation” OR “myofascial pain” OR “trigger points” OR “neural mechanosensitivity” OR “radicular symptoms” OR “biomechanics”)
Scopus	January 1990 to June 2026	TITLE-ABS-KEY (“chronic neck pain” OR “non-specific neck pain” OR “upper thoracic pain”) AND TITLE-ABS-KEY (“forward head posture” OR “cervical flexion” OR “thoracic posture” OR “postural loading” OR “sedentary behaviour” OR “smartphone” OR “computer work”) AND TITLE-ABS-KEY (“muscle stiffness” OR “muscle tone” OR “muscle fatigue” OR “myotonometry” OR “MyotonPRO” OR “shear-wave elastography” OR “electromyography”) AND TITLE-ABS-KEY (“microcirculation” OR “oxygenation” OR “myofascial pain” OR “trigger points” OR “neural mechanosensitivity” OR “radicular symptoms” OR “biomechanics”)
Web of Science	January 1990 to June 2026	TS = (“chronic neck pain” OR “non-specific neck pain” OR “upper thoracic pain”) AND TS = (“forward head posture” OR “cervical flexion” OR “thoracic posture” OR “postural loading” OR “sedentary behaviour” OR “smartphone” OR “computer work”) AND TS = (“muscle stiffness” OR “muscle tone” OR “muscle fatigue” OR “myotonometry” OR “MyotonPRO” OR “shear-wave elastography” OR “electromyography”) AND TS = (“microcirculation” OR “oxygenation” OR “myofascial pain” OR “trigger points” OR “neural mechanosensitivity” OR “radicular symptoms” OR “biomechanics”)
Supplementary targeted search	January 1990 to June 2026	“central sensitization,” “central sensitisation,” “nociplastic pain,” “pain hypersensitivity,” “hyperalgesia,” “allodynia,” “pain neuroscience,” and “pain neuroscience education”
